# Longitudinal Associations of Rest-Activity Rhythm Profiles with Perceived Physical Fatigability

**DOI:** 10.1093/geroni/igaf122.3806

**Published:** 2025-12-31

**Authors:** Namhyun Kim, Terri Blackwell, Katie Stone, Reagan Garcia, Peggy Cawthon, Melissa Erickson, Karyn Esser, Nancy Glynn

**Affiliations:** University of Pittsburgh, Pittsburgh, Pennsylvania, United States; California Pacific Medical Center Research Institute, San Francisco, California, United States; California Pacific Medical Center Research Institute, Castro Valley, California, United States; University of Pittsburgh, Pittsburgh, Pennsylvania, United States; California Pacific Medical Center, San Francisco, California, United States; AdventHealth, Orlando, Florida, United States; University of Florida College of Medicine, Gainesville, Florida, United States; University of Pittsburgh, Pittsburgh, Pennsylvania, United States

## Abstract

Rest-activity rhythms (RARs), 24-hour activity and sleep patterns, are cross-sectionally associated with fatigability, a prognostic marker of functional decline. Participants (N = 821, age=76.4±5.0 years) from the Study of Muscle, Mobility and Aging had perceived physical fatigability measured using the validated 10-item Pittsburgh Fatigability Scale (PFS; 0-50, higher score=greater fatigability) at baseline and up to three follow-up visits over 2.6±0.4 years. RARs were derived from 7-day wrist-worn accelerometry using the extended cosine model (parametric: amplitude, acrophase, alpha, beta) or non-parametric approaches (interdaily stability, intradaily variability, relative amplitude). Three RAR profiles (Synchronized, Concentrated High-Activity, Low-Fragmented) were identified using the k-means clustering algorithm. Compared to Synchronized, the Concentrated High-Activity group had the highest amplitude (5,176 vs. 2,258 counts/min) and earliest acrophase (11:54AM vs. 2:18PM). The Low-Fragmented group vs. Synchronized had the lowest amplitude (1,462 vs. 2,258 counts/min), interdaily stability (0.46 vs. 0.63), and highest intradaily variability (1.10 vs. 0.81). PFS score varied by RAR groups: Synchronized=15.2±8.5, Concentrated High-Activity=14.2±7.9, and Low-Fragmented=17.3±8.7, p = 0.004. Using linear mixed models, the Low-Fragmented group had higher perceived physical fatigability at baseline (β = 1.30, p = 0.046) and a steeper increase in PFS score over time (β = 0.46, p = 0.03) vs. Synchronized, adjusted for demographics, health conditions, lifestyle factors, and sleep efficiency. After further adjustment for cardiorespiratory fitness (VO2peak from treadmill testing) and daily step count, the Low-Fragmented group showed a significantly larger increase in PFS score over time (β = 0.46, p = 0.03; β = 0.49, p = 0.02), although the baseline association was attenuated. Our findings reveal that disrupted RARs are independently associated with persistently worsening perceived physical fatigability.

